# Beyond Resistance Genes: *Pseudomonas aeruginosa* as a Complex Adaptive System Driving Persistence, Evolution, and Antimicrobial Resistance

**DOI:** 10.3390/life16071163

**Published:** 2026-07-14

**Authors:** Ayman Elbehiry, Eman Marzouk

**Affiliations:** Department of Public Health, College of Applied Medical Sciences, Qassim University, P.O. Box 6666, Buraydah 51452, Saudi Arabia; ar.elbehiry@qu.edu.sa

**Keywords:** *Pseudomonas aeruginosa*, antibiotic resistance, complex adaptive systems, adaptive resilience cascade, phenotypic plasticity, persistence, biofilms, population heterogeneity, evolution, systems biology

## Abstract

*Pseudomonas aeruginosa* (*P. aeruginosa*) is one of the most adaptable bacterial pathogens and a major cause of difficult-to-treat infections worldwide. Although antimicrobial resistance (AMR) is commonly attributed to resistance genes and their associated mechanisms, this perspective does not fully explain the ability of *P. aeruginosa* to survive antimicrobial exposure, establish chronic infections, and persist across diverse environmental and host-associated habitats. In this review, we examine *P. aeruginosa* within a complex adaptive systems framework, integrating evidence from molecular microbiology, physiology, ecology, population biology, and evolutionary genomics. We describe how environmental sensing, regulatory integration, phenotypic plasticity, population heterogeneity, persistence, biofilm formation, collective behavior, and evolutionary diversification interact across biological scales to shape bacterial survival and long-term success. Evidence from chronic infections and environmental reservoirs indicates that resistance emerges from interconnected physiological, ecological, and evolutionary processes rather than from isolated genetic determinants alone. Building on these observations, we propose an adaptive resilience cascade framework in which environmental sensing drives physiological diversification, persistence maintains survival under stress, evolutionary selection stabilizes advantageous traits, and ecological dissemination promotes the spread of successful lineages. This framework provides a systems-level explanation for treatment failure, chronic colonization, and resistance emergence while linking cellular responses to population, ecological, and evolutionary outcomes. Emerging approaches, including single-cell analyses, spatial omics, evolution-informed interventions, engineered biological therapeutics, and artificial intelligence–assisted modeling, further support a shift toward targeting adaptive resilience rather than resistance determinants alone. Viewing *P. aeruginosa* as a complex adaptive system offers an integrated conceptual foundation for future surveillance, therapeutic development, and antimicrobial stewardship strategies.

## 1. Introduction

Antimicrobial resistance (AMR) is a growing global threat that is reducing the effectiveness of antimicrobial therapies and increasing the burden of healthcare-associated infections. Recent estimates indicate that bacterial AMR contributes to millions of deaths each year and imposes substantial clinical and economic costs on healthcare systems worldwide [1,2]. Despite advances in antimicrobial stewardship, infection control, and drug development, the burden of resistant infections is expected to increase in the coming decades [2]. Among the bacterial pathogens prioritized by the World Health Organization, *Pseudomonas aeruginosa* (*P. aeruginosa*) remains a major concern because of its extensive resistance profile, frequent occurrence in healthcare settings, and exceptional ability to survive in diverse environmental and host-associated niches [3,4]. As a leading cause of healthcare-associated infections, chronic respiratory infections, bloodstream infections, and device-associated infections, *P. aeruginosa* exemplifies the challenge posed by highly adaptable bacterial pathogens [5,6].

Research on AMR has traditionally focused on resistance genes and their molecular mechanisms [7]. Although this approach has greatly improved our understanding of resistance acquisition and dissemination, it does not fully explain the behavior of *P. aeruginosa* during infection [8,9]. Clinical persistence and treatment failure can occur even when isolates remain susceptible in laboratory testing, indicating that additional factors contribute to bacterial survival [10,11]. Antibiotic tolerance, persister-cell formation, biofilm-associated protection, and physiological adaptation can allow bacterial populations to survive antimicrobial exposure without acquiring stable resistance mutations [9,12,13]. These observations indicate that resistance in *P. aeruginosa* is not solely a genetic trait. Instead, it arises from interactions among genetic determinants, physiological states, population structure, and environmental conditions that collectively shape bacterial survival and treatment outcomes [8,14].

The ecological and clinical success of *P. aeruginosa* reflects its ability to function as a highly adaptable biological system. Unlike many pathogens that occupy relatively narrow ecological niches, *P. aeruginosa* thrives across a wide range of environmental and host-associated habitats [15,16]. This adaptability is supported by extensive metabolic flexibility, complex regulatory networks, and sophisticated environmental sensing systems that enable rapid responses to changing conditions [17,18]. These networks integrate signals related to nutrient availability, population density, host-derived stress, oxidative stress, and antimicrobial exposure, coordinating changes in metabolism, physiology, motility, virulence, and community behavior [19,20]. Through these interconnected processes, *P. aeruginosa* continuously adjusts its behavior, linking environmental sensing, physiological adaptation, and evolutionary change within a unified adaptive system [8,21].

A key feature of this adaptive capacity is phenotypic plasticity. In response to environmental variation, genetically related cells can adopt distinct physiological and behavioral states, generating substantial diversity within a single population [22,23]. This flexibility promotes population heterogeneity, allowing subpopulations to exploit different resources, tolerate stress, and occupy distinct microenvironments [24,25]. During chronic infections, particularly in the cystic fibrosis airway, prolonged exposure to heterogeneous selective pressures drives the emergence of variants that differ in metabolism, virulence, motility, and antimicrobial susceptibility [25,26]. Among these variants are persister cells, a specialized subpopulation capable of surviving intense antimicrobial stress through transient dormancy and extreme tolerance [27,28]. Together, phenotypic plasticity, population heterogeneity, and persistence provide a reservoir of adaptive potential that enhances resilience, supports long-term survival, and promotes successful colonization across diverse ecological niches [23,29].

Despite major advances in understanding resistance mechanisms, virulence, biofilm formation, persistence, and evolution, these processes are often studied independently. As a result, the interactions among ecological pressures, regulatory networks, phenotypic variation, persistence, and population dynamics remain incompletely understood [8,30]. Emerging systems-level studies show that many clinically important traits of *P. aeruginosa* arise from interactions across molecular, cellular, population, and environmental scales rather than from isolated genetic determinants alone [31]. An integrative framework is therefore needed to explain how adaptation, persistence, and resistance emerge across biological scales.

In this review, we present *P. aeruginosa* as a dynamic system that adapts, persists, and evolves in response to changing conditions. Unlike previous reviews, which mainly discuss AMR, biofilms, persistence, virulence, or evolution separately, we bring these processes together within a single complex adaptive systems framework. We also introduce the adaptive resilience cascade, a conceptual model that explains how environmental sensing, phenotypic plasticity, persistence, evolution, and ecological spread work together to promote long-term survival and AMR. By linking these processes across different biological levels, this review provides a broader understanding of treatment failure, chronic infection, and resistance development, while highlighting new opportunities for surveillance, therapeutic development, and antimicrobial stewardship.

## 2. Literature Search Strategy

This narrative review was developed through a comprehensive literature search to identify studies relevant to the adaptation, persistence, ecological success, evolution, and AMR of *P. aeruginosa*. Searches were conducted in PubMed, Scopus, Web of Science, and Google Scholar using combinations of keywords including “*P. aeruginosa*”, AMR, persistence, tolerance, biofilms, phenotypic plasticity, population heterogeneity, quorum sensing, evolution, adaptation, ecological reservoirs, systems biology, and complex adaptive systems.

The search emphasized literature published between 2000 and 2026 to capture current advances in microbial ecology, evolutionary biology, systems microbiology, and AMR research. Earlier landmark studies were included when they provided foundational insights into environmental sensing, regulatory adaptation, biofilm biology, persistence, population dynamics, or evolutionary processes that remain central to current understanding.

Eligible sources included peer-reviewed original research articles, systematic reviews, meta-analyses, and authoritative reports published in English. Studies were selected according to their relevance to the major themes addressed in this review, including environmental sensing, phenotypic plasticity, regulatory adaptation, population heterogeneity, persistence mechanisms, biofilm-associated survival, collective behavior, ecological dissemination, evolutionary dynamics, and resistance emergence. Additional relevant publications were identified through manual screening of reference lists from key articles.

Publications were excluded if they were not directly related to the objectives of the review, focused primarily on microorganisms other than *P. aeruginosa*, lacked sufficient scientific detail, were conference abstracts without accompanying full-text publications, or represented duplicate reports of previously published findings. Because this study is a narrative review, formal systematic-review procedures, risk-of-bias assessment, and quantitative meta-analytic methods were not applied. Instead, evidence from multiple disciplines was critically synthesized to develop an integrated systems-level perspective of *P. aeruginosa* adaptation, persistence, and AMR.

Because this is a narrative review, the literature was selected to provide a balanced overview of the major biological processes involved in adaptation and AMR rather than to compare specific bacterial lineages or ecological niches. Both clinical and environmental studies were considered when they contributed to understanding the systems-level behavior of *P. aeruginosa*. Nonetheless, studies of clinically important isolates, particularly those from chronic infections, are more abundant in the published literature and are therefore more strongly represented.

## 3. *P. aeruginosa* as a Complex Adaptive System

### 3.1. Principles of Complex Adaptive Systems in Microbiology

Complex adaptive systems (CAS) are composed of interacting elements whose collective properties arise from continuous exchanges among components rather than from the actions of individual units alone. These systems are characterized by distributed control, feedback regulation, nonlinear responses, self-organization, and the capacity to adjust to changing circumstances. As a result, system-level behaviors often cannot be explained by examining isolated components. Increasing evidence indicates that microorganisms exhibit many of these characteristics because cellular activities reflect the integration of genetic, physiological, ecological, and environmental influences operating across multiple organizational levels. From this perspective, bacterial populations function as coordinated biological networks that detect external change, modify collective activities, and maintain functionality under fluctuating conditions [21,30]. In this review, the CAS framework serves as an evidence-based model supported by findings from regulatory, ecological, physiological, and evolutionary studies rather than as a purely theoretical construct. Within this framework, adaptive resilience refers to the capacity of bacterial populations to preserve survival, functionality, and evolutionary potential despite environmental, host-associated, or antimicrobial challenges.

Among bacterial pathogens, *P. aeruginosa* provides a particularly valuable model for examining microbial complexity. This opportunistic species occupies a broad range of habitats, including soil, aquatic ecosystems, plants, animals, and human hosts, while retaining the ability to cause both acute and chronic disease. Its ecological success reflects exceptional metabolic versatility, extensive regulatory capabilities, and a remarkable ability to function across diverse settings [21,32]. Instead of responding through isolated molecular pathways, *P. aeruginosa* coordinates information from multiple external signals through integrated regulatory and metabolic networks. Consequently, its behavior reflects the combined influence of cellular regulation, metabolism, population structure, and ecological context rather than the activity of individual genes alone.

A central feature of CAS is the ability to convert environmental information into coordinated biological outcomes. In *P. aeruginosa*, this function relies on interconnected sensory and regulatory pathways that detect nutrient availability, oxygen levels, osmotic stress, host-derived signals, oxidative stress, and antimicrobial exposure [33]. Two-component regulatory systems, alternative sigma factors, and other transcriptional regulators rapidly modify gene expression in response to changing conditions. Early evidence for this capacity came from studies of AlgR and related environmentally responsive regulators that coordinate bacterial behavior within host-associated environments [34]. Subsequent work demonstrated that local conditions strongly influence quorum sensing (QS) activity and downstream transcriptional programs, emphasizing the context-dependent nature of bacterial decision-making [35]. Additional studies showed that QS signals can be integrated with other sensory inputs, including light-dependent pathways, allowing coordinated population responses to complex environmental stimuli [36].

The ability of *P. aeruginosa* to process and integrate diverse signals depends on an extensive regulatory architecture. Instead of operating through simple linear pathways, the organism relies on interconnected networks of transcription factors, sigma factors, QS regulators, and two-component systems that collectively govern cellular functions [17]. Systems-level analyses have revealed hundreds of regulatory interactions controlling virulence, metabolism, biofilm development, stress responses, and niche-specific behavior [17,20]. This architecture displays hierarchical organization, functional redundancy, and extensive cross-talk between regulatory modules. Such features preserve operational stability while allowing rapid responses to external disturbances. Within this network, QS functions as one component of a broader regulatory landscape rather than as an independent communication system, linking population-level signaling with physiological and environmental inputs [37,38].

Another defining characteristic of CAS is the appearance of higher-order properties that arise from local interactions among individual components. In *P. aeruginosa*, these properties are particularly evident during biofilm formation and community-level organization. Biofilms are structured multicellular assemblies whose behavior cannot be predicted solely from the characteristics of free-living cells. Interactions involving cell-to-cell communication, extracellular matrix production, metabolic cooperation, and spatial organization generate collective properties that improve survival under unfavorable conditions [39]. Biofilm development is directed by coordinated signaling pathways that regulate attachment, maturation, maintenance, and dispersal according to local conditions [40]. Likewise, interactions among neighboring cells can promote self-organization and generate complex spatial arrangements, demonstrating how population-level structure can arise from relatively simple cellular processes [41].

Population diversity represents another key feature of complex systems [21,23]. Although bacterial populations are often viewed as genetically uniform, individual cells frequently differ in physiology, metabolism, stress responses, and communication patterns [23,42]. This variability produces distinct cellular states that contribute differently to growth and survival. Recent work has shown that QS activity can become temporarily partitioned among subpopulations, creating heterogeneous communication patterns that influence collective decision-making [43]. Such variation broadens the range of available survival strategies within a population and increases resilience when conditions change. Consequently, population behavior reflects the combined contributions of multiple functionally distinct subgroups rather than a uniform response shared by all cells [23,42,43].

Support for viewing *P. aeruginosa* as a CAS has been strengthened by advances in systems biology. Genome-scale metabolic reconstructions, transcriptomic studies, network analyses, and multi-omics approaches consistently demonstrate that many clinically important phenotypes arise from interactions among regulatory, metabolic, and ecological processes [30]. Similarly, systems-level investigations of AMR indicate that bacterial responses involve coordinated shifts in metabolism, regulatory circuitry, and cellular function that extend beyond classical resistance determinants [44]. These observations challenge reductionist interpretations and highlight the importance of examining biological processes across multiple organizational levels.

**Figure 1:**
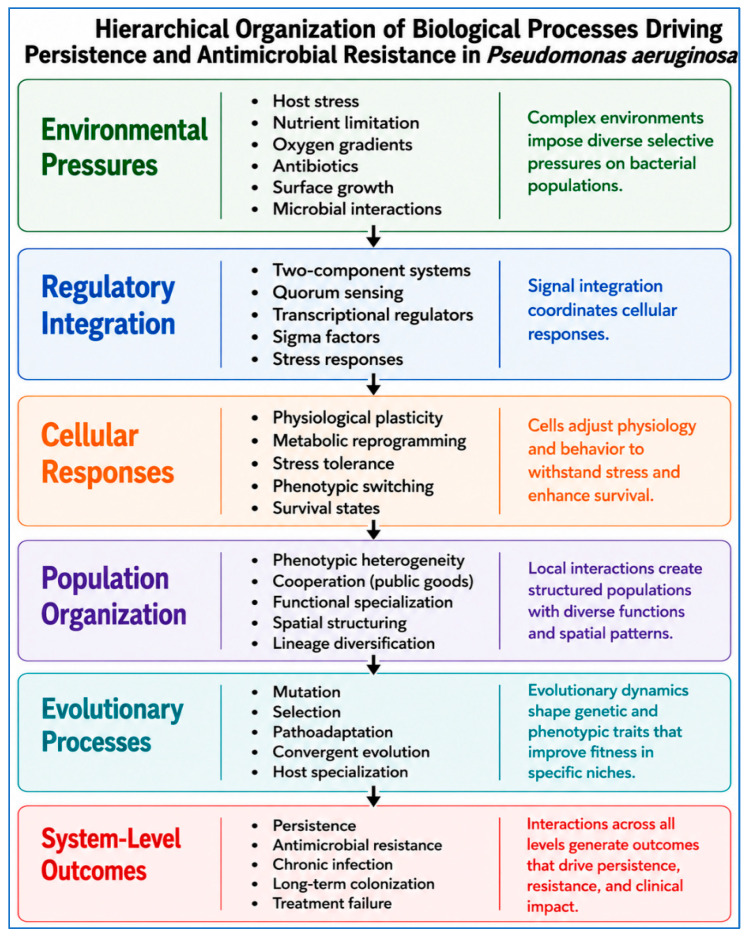
Hierarchical organization of biological processes underlying the ecological success and persistence of *P. aeruginosa*. Environmental pressures are integrated through regulatory networks that shape cellular responses, population organization, and evolutionary trajectories. Interactions across these levels generate system-level outcomes, including persistence, AMR, chronic infection, ecological success, and treatment recalcitrance.

Collectively, available evidence indicates that *P. aeruginosa* displays the major attributes of a complex adaptive system. Environmental perception, regulatory integration, self-organization, collective behavior, cellular diversity, and systems-level coordination combine to promote ecological success, long-term persistence, and pathogenicity [17,20,21,30]. Viewing *P. aeruginosa* through this lens provides a foundation for understanding how resistance, persistence, virulence, and evolutionary change arise from linked processes operating across hierarchical levels of biological organization [21,30,45,46]. This framework also provides the basis for examining emergent resistance and multiscale responses discussed in the following section. The relationships among these processes are summarized in Figure 1.

### 3.2. Emergent Resistance and Multi-Scale Adaptation

Resistance in *P. aeruginosa* is increasingly understood as a system-level outcome arising from interactions among genetic, physiological, ecological, and evolutionary processes. Rather than resulting solely from the acquisition of specific resistance determinants, resistance develops through continuous exchanges between bacterial populations and their surroundings. This perspective helps explain why resistance phenotypes can arise rapidly under selection and differ considerably across clinical and environmental settings [21,47,48,49].

At the cellular level, *P. aeruginosa* displays pronounced physiological flexibility that supports survival under changing circumstances [8,21]. Nutrient limitation, oxidative stress, host immune activity, antimicrobial exposure, and physicochemical gradients trigger extensive transcriptional changes that reshape metabolism and cellular function [48,49,50]. These adjustments can produce temporary states associated with increased tolerance to antimicrobial stress without requiring stable genetic alterations [8,48]. Environmental sensing pathways therefore serve as key links between external challenges and intracellular survival programs, enabling rapid responses before resistance mutations become established [21,48].

Local heterogeneity further influences bacterial behavior during infection. Within host tissues and structured microbial communities, gradients of oxygen, nutrients, metabolites, signaling molecules, and antimicrobial concentrations create distinct niches that support diverse cellular states [25,51]. Early experimental work demonstrated that oxygen availability generates marked spatial variation within *P. aeruginosa* biofilms, confining highly active cells to specific regions while neighboring cells experience substantially different physiological conditions [52]. As a result, populations sharing the same genetic background can respond differently to both host-derived and therapeutic pressures [25,51,52].

Recent advances in spatial transcriptomics and multiscale modeling have provided further insight into this complexity. Localized physicochemical conditions drive extensive metabolic diversification within *P. aeruginosa* communities, allowing distinct subpopulations to occupy aerobic, hypoxic, denitrifying, oxidative stress-associated, or alternative metabolic states simultaneously [51,53]. This functional specialization indicates that population behavior cannot be fully understood by examining individual cells alone. Instead, community-level outcomes arise from interactions among diverse cellular subgroups and the microenvironments they inhabit [24,25]. These explanations reinforce the view that bacterial physiology, spatial organization, and local habitat conditions are closely linked determinants of survival and fitness [43,51,53].

Cellular diversity also expands the range of phenotypes available for selection, promoting evolutionary diversification over time [23,24,25]. During prolonged stress, bacterial populations accumulate mutations that improve performance within specific niches [25,49]. Long-term colonization is therefore accompanied by continuous genetic and phenotypic remodeling [26,49]. Hypermutable lineages, recurrent pathoadaptive mutations, and convergent evolutionary patterns have been repeatedly documented in chronic infections, demonstrating how selective forces shape population structure during persistence [25,54,55]. Antibiotic exposure can also enrich mutator populations, accelerating diversification and increasing the likelihood that resistant variants will arise [56].

Host-associated evolution represents another important component of resistance development. During chronic infection, *P. aeruginosa* undergoes extensive changes in regulatory, metabolic, and stress-response pathways that support long-term survival within host tissues [21,26,54]. Comparative genomic studies have identified recurrent host-specific evolutionary trajectories and pathoadaptive genes associated with successful colonization [49]. These findings indicate that resistance frequently develops alongside broader biological changes that enhance fitness within particular host niches [8,49].

Selective forces originating from the host and surrounding environment further shape evolutionary outcomes. Oxidative stress, nutrient deprivation, antimicrobial exposure, and surface-associated growth activate distinct regulatory programs that influence survival and long-term population trajectories [21,49,50]. Importantly, these pressures rarely occur independently. Instead, bacterial behavior reflects the combined influence of multiple concurrent challenges [8,21]. Studies of adaptive resistance have shown that specific environmental cues can induce temporary resistance phenotypes that disappear once the initiating stimulus is removed, underscoring the context-dependent nature of bacterial responses [48,50].

Clinical observations support the view that resistance is an ongoing evolutionary process rather than a fixed endpoint. Resistance emerging during treatment has been documented following exposure to multiple antipseudomonal agents, illustrating how therapeutic interventions can function as powerful ecological filters that reshape population composition [57,58]. Whether resistant variants become established depends not only on the antimicrobial agent used but also on population diversity, local conditions, and the biological characteristics of the infecting strain. These findings illustrate that resistance in *P. aeruginosa* arises through processes operating across cellular, population, community, and host levels [8,47]. Physiological flexibility, population diversity, ecological heterogeneity, and evolutionary change combine to generate resistance phenotypes that cannot be fully explained by individual genes or mechanisms alone [21,24,25]. This broader perspective provides a more complete explanation for the ability of *P. aeruginosa* to withstand antimicrobial intervention and establish persistent infections [8,43,51].

Building on these observations, we propose that resistance develops through an adaptive resilience cascade in which environmental sensing initiates physiological diversification, persistence preserves survival during stress, evolutionary selection stabilizes advantageous traits, and ecological dissemination promotes the spread of highly successful lineages. Within this framework, AMR is viewed as one outcome of a broader biological process operating across multiple levels of organization [8,21,30,45]. Although supported by evidence from ecological, physiological, evolutionary, and systems-level investigations, this framework should currently be considered a conceptual synthesis rather than a validated predictive model.

To establish this framework as a predictive model, future studies should examine whether changes in environmental conditions consistently lead to measurable changes in regulatory activity, phenotypic diversity, persistence, and evolutionary adaptation across different experimental and clinical settings. Combining longitudinal clinical studies with single-cell analyses, spatial omics, multi-omics approaches, and mathematical modeling will help determine whether the proposed adaptive resilience cascade can reliably predict bacterial behavior and treatment outcomes [30,31,44]. Figure 1 illustrates how these processes interact across hierarchical levels to shape resistance and long-term survival.

### 3.3. From Individual Cells to Population-Level Dynamics

The success of *P. aeruginosa* depends not only on the behavior of individual cells but also on the capacity of populations to generate, maintain, and organize biological diversity across space and time. As bacterial communities develop, they become structured assemblies of interacting subpopulations whose collective properties extend beyond those of individual members. Thus, long-term ecological and evolutionary outcomes are increasingly shaped by population-level processes that influence resource utilization, niche occupation, and survival [21,23].

One consequence of sustained selection is ecological diversification. Experimental studies have shown that populations originating from a single ancestral *Pseudomonas* lineage can rapidly diversify into specialized variants occupying distinct niches through adaptive radiation [59]. Rather than converging on a single optimal phenotype, evolving populations often produce multiple successful strategies that coexist within the same environment [23,59]. This diversification broadens resource utilization, reduces direct competition among lineages, and increases the overall ecological range available to the population [59].

Cooperative interactions further strengthen population organization. Many traits that contribute to ecological success depend on extracellular products that influence neighboring cells and modify local surroundings. Pyoverdine production provides a well-characterized example of such cooperation, with expression levels varying according to ecological context and population structure [22]. Through these shared activities, bacterial communities collectively acquire resources and sustain functions that would be less efficient at the level of isolated cells. The benefits generated by cooperation contribute to community stability and support long-term population persistence [22,23].

Interactions among neighboring cells can also produce complex spatial organization. Gelimson et al. [41] demonstrated that *P. aeruginosa* cells form multicellular structures through interactions with secreted polymeric trails, generating organized communities without centralized control. These self-generated spatial arrangements influence movement, local communication, and resource accessibility, illustrating how simple cellular interactions can shape large-scale population architecture.

The importance of these collective processes becomes particularly apparent during chronic host colonization. Long-term infections expose bacterial populations to diverse and persistent selective forces that promote lineage diversification and evolutionary experimentation. Genomic studies of *P. aeruginosa* recovered from cystic fibrosis patients have revealed extensive within-host diversity characterized by the coexistence of multiple evolutionary lineages [54,55]. Instead of being dominated by a single successful clone, chronic populations often consist of distinct sublineages that accumulate independent genetic changes while persisting within the same host environment.

Notably, these coexisting lineages frequently acquire similar traits despite following separate evolutionary paths. Comparative genomic analyses have identified recurrent mutations affecting regulatory functions, metabolism, AMR, and host-associated fitness, providing strong evidence of convergent evolution during chronic infection [54]. At the same time, substantial genetic divergence is maintained among subpopulations, indicating that multiple successful solutions can coexist under the selective pressures present within the host. Feliziani et al. [55] showed that diversified lineages may persist together for many years, producing complex population structures shaped by ongoing mutation and selection.

These observations indicate that the evolutionary behavior of *P. aeruginosa* cannot be fully explained by examining individual cells or isolated genetic event [54,55,59]. Over time, bacterial communities function as dynamic evolutionary networks composed of interacting and diverging lineages that collectively explore alternative trajectories. Understanding these population-level processes provides an important bridge between cellular behavior and the broader ecological and evolutionary patterns that contribute to long-term persistence. The relationships linking environmental pressures, regulatory coordination, cellular activities, population organization, and system-level outcomes are summarized in Figure 1.

## 4. Environmental Sensing and Phenotypic Plasticity

Phenotypic plasticity allows *P. aeruginosa* to alter cellular functions in response to local ecological cues. As a result, genetically related cells can differ in metabolism, stress tolerance, behavior, and resource utilization despite sharing the same genome. This capacity supports survival across diverse habitats and contributes to the remarkable ecological breadth of the species [42].

### 4.1. Environmental Sensing and Regulatory Adaptation

Environmental information is translated into coordinated cellular responses through an extensive network of sensory and regulatory pathways. These systems monitor oxygen availability, nutrient status, iron concentration, oxidative stress, and surface association, enabling rapid adjustments in gene expression and cellular function [33]. Oxygen availability is one of the most influential determinants of bacterial physiology. Within biofilms and infected tissues, steep oxygen gradients generate niches ranging from fully aerobic to nearly anoxic environments [52,60]. Responses to oxygen limitation are controlled primarily by the transcriptional regulator Anr, which governs genes required for growth under microoxic and anoxic conditions [60]. When oxygen becomes scarce, Anr activates the denitrification regulator Dnr, allowing the use of alternative respiratory pathways that sustain energy production under oxygen-restricted conditions [60].

Iron availability provides another critical source of regulatory input. Because free iron is tightly restricted within host tissues, *P. aeruginosa* relies on the ferric uptake regulator Fur to maintain iron homeostasis. Fur coordinates siderophore biosynthesis, iron transport systems, and several virulence-associated functions according to intracellular iron status [61]. Through the controlled production of pyoverdine and pyochelin, bacterial cells maximize iron acquisition while limiting the toxic effects of iron overload [61].

Host-derived oxidative stress activates protective pathways centered on OxyR and related antioxidant regulators. These systems induce catalases, peroxidases, and hydroperoxide-detoxifying enzymes that limit oxidative damage and promote survival during infection [62]. Surface attachment initiates another major regulatory transition. Elevated cyclic-di-GMP levels stimulate biofilm development, extracellular matrix production, and reduced motility, supporting the transition from a free-living to a surface-associated mode of growth [63]. These regulatory pathways connect external signals to coordinated cellular programs that shape bacterial behavior in diverse ecological and host-associated settings.

### 4.2. Phenotypic Heterogeneity and Metabolic Flexibility

One consequence of regulatory coordination is the generation of phenotypic heterogeneity. Rather than responding uniformly to external stimuli, *P. aeruginosa* populations often contain subgroups with distinct functional characteristics [42]. Within biofilms, gradients of oxygen and nutrients create localized niches that support different patterns of cellular activity [52]. Using microelectrode and reporter-based approaches, Xu et al. [52] demonstrated that oxygen availability produces pronounced stratification within biofilms, with active protein synthesis concentrated near oxygenated surface regions. Cells located deeper within the biofilm experience markedly different metabolic environments despite sharing the same genetic background.

These localized differences generate functionally distinct cellular subpopulations and contribute to community-level variation [51]. The resulting organization highlights how spatially structured habitats shape bacterial behavior within a single microbial community. Such variation is reinforced by the exceptional metabolic versatility of *P. aeruginosa*. The organism can utilize a wide range of carbon sources, switch between aerobic and anaerobic metabolism, and exploit alternative electron acceptors when resources become limited. This metabolic breadth supports growth across diverse niches and facilitates survival under fluctuating conditions [60].

**Figure 2:**
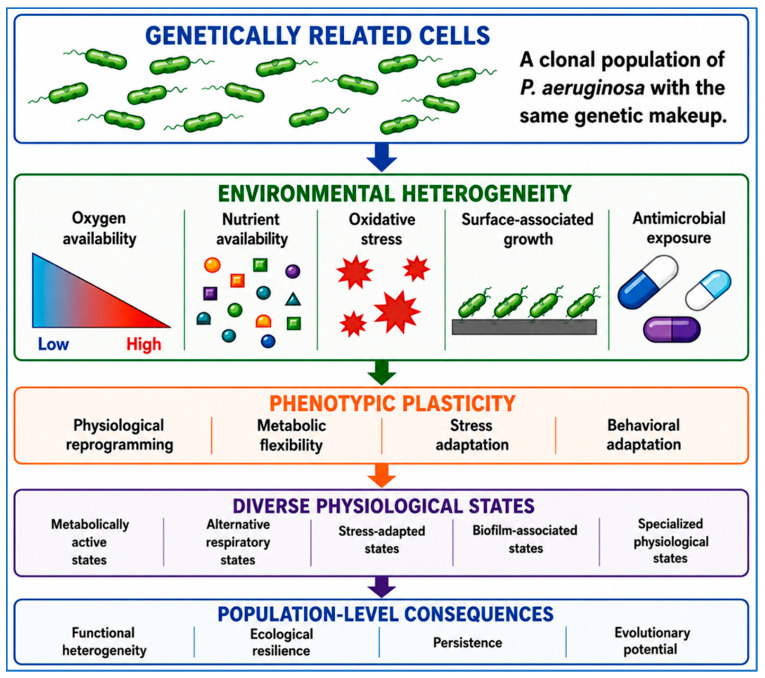
Environmental heterogeneity promotes phenotypic plasticity in *P. aeruginosa*. Variation in local ecological conditions triggers reversible physiological adjustments that allow genetically related cells to adopt distinct functional states. The resulting cellular diversity contributes to population heterogeneity, persistence, ecological resilience, and long-term evolutionary potential.

Variation within populations extends beyond metabolism. Individual cells may differ in stress tolerance, QS activity, virulence factor production, motility, participation in biofilm development, and antimicrobial susceptibility [42,43]. These differences generate functionally specialized subpopulations that contribute to the overall resilience and persistence of the community. Through the combined effects of environmental sensing, regulatory coordination, and metabolic flexibility, *P. aeruginosa* produces a spectrum of cellular phenotypes that enhance survival across diverse ecological settings [42,43]. The relationship between environmental heterogeneity, phenotypic plasticity, and the emergence of diverse physiological states is summarized in Figure 2.

### 4.3. Plasticity as a Driver of Survival and Adaptation

Phenotypic plasticity enables *P. aeruginosa* to respond rapidly to changing conditions without requiring immediate genetic change. Through reversible shifts in cellular function, bacterial populations can cope with nutrient limitation, oxidative stress, host immune activity, and antimicrobial exposure [23,42]. In the short term, these adjustments promote survival by generating transient cellular programs that improve tolerance to adverse conditions. Changes in metabolism, growth dynamics, and stress-response pathways can increase survival during antibiotic treatment and other environmental challenges before stable genetic alterations become established [48].

At the population level, the coexistence of diverse cellular phenotypes functions as a form of biological risk management. By maintaining multiple functional strategies simultaneously, bacterial communities reduce the likelihood that a single stressor will eliminate the entire population. As a result, heterogeneous populations often withstand environmental disturbances more effectively than populations that respond uniformly [42].

Plasticity may also shape long-term evolutionary trajectories. Spatially structured habitats support multiple cellular phenotypes that experience different selective pressures at the same time. Chronic infections provide a well-documented example of this process. Within the host, heterogeneous microenvironments promote the emergence and persistence of genetically distinct sublineages occupying different ecological niches [54]. These conditions create opportunities for parallel evolutionary pathways, allowing multiple lineages to explore alternative solutions to common challenges while remaining within the same population.

By enabling rapid physiological adjustment while preserving functional diversity, phenotypic plasticity strengthens the capacity of *P. aeruginosa* to persist in fluctuating environments. This flexibility supports both immediate survival and longer-term evolutionary diversification, reinforcing the ecological success of the species across a wide range of host-associated and environmental settings. The major environmental signals, cellular responses, and ecological consequences discussed in this section are summarized in Table 1.

## 5. Persistence and Biofilms as Integrated Survival Strategies

The long-term survival of *P. aeruginosa* in environmental and host-associated settings depends on mechanisms that extend beyond genetically encoded AMR. Among the most important are persistence and biofilm formation, two complementary processes that enable bacterial populations to withstand antimicrobial exposure, host defenses, and fluctuating ecological pressures. Although often discussed independently, these phenomena are closely linked and play central roles in chronic colonization and treatment failure [16].

### 5.1. Persistence, Tolerance, and Transient Survival States

The ability of *P. aeruginosa* to survive antimicrobial treatment cannot be explained solely by resistance mutations. Bacterial populations can enter reversible phenotypic programs that reduce susceptibility to killing without altering minimum inhibitory concentrations. These non-heritable responses include tolerance, persistence, adaptive resistance, and related phenotypic variants that collectively improve survival during environmental and therapeutic stress [9,64]. Resistance permits bacterial growth in the presence of antibiotics, tolerance prolongs survival during exposure, whereas persistence reflects the survival of specialized subpopulations that remain viable despite treatment [9,13,14].

Tolerance describes the ability of bacterial populations to remain viable during prolonged exposure to bactericidal antibiotics through physiological changes that slow antibiotic-mediated killing. Unlike resistant cells, tolerant cells do not proliferate in the presence of antimicrobial agents but instead survive until favorable conditions return. Nutrient limitation, oxygen restriction, starvation, and antimicrobial exposure can induce tolerant phenotypes that disappear once the initiating stimulus is removed [9,64].

In chronic cystic fibrosis infections, oxygen limitation is considered the primary environmental factor driving bacterial diversification within biofilms. Reduced oxygen availability creates distinct metabolic zones that promote slow growth, persistence, and specialized physiological states. Nutrient limitation further increases this diversity by shaping metabolic activity and stress responses in different regions of the biofilm. These environmental gradients generate the functional heterogeneity that supports long-term persistence and reduced susceptibility to antimicrobial treatment [13,28].

Persister cells represent a distinct subset of tolerant cells characterized by markedly reduced metabolic activity and growth. Because many antibiotics target active cellular processes, dormant or slow-growing cells can survive treatment while most of the population is eliminated. After antimicrobial pressure is removed, persisters can resume growth and regenerate a susceptible population, contributing to recurrent infection and treatment failure [65]. Clinical studies have documented the emergence of high-persister *P. aeruginosa* lineages during chronic cystic fibrosis infections, indicating that repeated antibiotic exposure can select for populations with an enhanced capacity to survive treatment even in the absence of stable resistance mechanisms [28].

Additional reversible responses further expand the range of survival strategies available to *P. aeruginosa*. Adaptive resistance arises in response to specific external stimuli and involves temporary changes in gene expression, membrane physiology, and antimicrobial susceptibility. Likewise, small-colony variants frequently recovered from chronic infections display altered metabolism, enhanced biofilm formation, increased matrix production, and elevated tolerance to multiple stresses. Increased cyclic-di-GMP signaling is commonly associated with these highly persistent phenotypes [9,21]. These findings reveal that *P. aeruginosa* employs multiple reversible mechanisms that allow populations to endure antimicrobial and host-associated challenges without immediate genetic change. These responses contribute to chronic infection and maintain reservoirs of viable cells that remain after antimicrobial treatment.

### 5.2. Biofilms as Structured Survival Niches

Biofilm formation is a defining feature of the *P. aeruginosa* lifestyle. Rather than existing exclusively as free-living cells, bacterial populations frequently organize into surface-associated communities embedded within a self-produced extracellular matrix. This transition creates highly organized multicellular structures that support long-term colonization and survival [21].

Biofilm development begins with surface sensing and attachment. Flagella, type IV pili, and multiple regulatory pathways contribute to the transition from reversible attachment to stable colonization. Subsequent production of extracellular polymeric substances, including polysaccharides, proteins, and extracellular DNA, establishes the structural framework of mature biofilms and promotes community stability [66]. Experimental studies demonstrated that biofilm maturation is controlled by coordinated signaling pathways that regulate progression through distinct developmental stages, emphasizing the highly organized nature of this process [40].

The importance of biofilms extends far beyond physical attachment. As biofilms mature, gradients of oxygen, nutrients, metabolites, and signaling molecules develop throughout the community, creating localized niches with distinct physicochemical characteristics. Classical physiological studies showed that oxygen is rapidly depleted within biofilms, restricting active growth to specific regions while deeper layers experience markedly different metabolic environments [52]. Accordingly, cells occupying different locations within the same biofilm encounter distinct selective pressures despite sharing the same genetic background.

Recent transcriptomic investigations have provided further evidence for this spatial organization. Mature *P. aeruginosa* biofilms contain subpopulations expressing distinct metabolic programs associated with aerobic respiration, denitrification, nitric oxide metabolism, and oxidative stress responses [51]. Local physicochemical variation therefore promotes functional specialization across the biofilm and generates a highly structured community. By creating diverse niches within a confined space, biofilms support multiple modes of growth, metabolism, and stress response that contribute substantially to long-term survival in both environmental and clinical settings.

### 5.3. Interactions Between Persistence and Biofilm-Mediated Protection

Persistence and biofilm formation reinforce one another during chronic colonization. Although biofilms provide protection at the community level, many of their protective properties arise from the heterogeneous conditions that develop within the biofilm itself. These conditions promote the formation and maintenance of transient phenotypes associated with reduced susceptibility to antimicrobial killing [9,13]. Within mature biofilms, oxygen limitation, nutrient restriction, and metabolic stratification generate subpopulations that differ markedly in physiological activity. Slow-growing and metabolically restricted cells are inherently less vulnerable to many antimicrobial agents because antibiotic activity often depends on active cellular processes. As a result, biofilms frequently harbor viable subpopulations that survive treatment despite lacking genetically encoded resistance mechanisms [13,52].

Spatial organization strengthens this effect by supporting the coexistence of multiple functional groups within the same community. Metabolically active cells, denitrifying populations, stress-responsive cells, and matrix-associated cells may occupy different regions of the biofilm, increasing the probability that a fraction of the population survives changing ecological or therapeutic pressures. Transcriptomic studies support this view by demonstrating extensive metabolic specialization within mature biofilms [51]. Protection is enhanced by the extracellular matrix, which can impede antimicrobial penetration, alter local chemical conditions, and buffer cells against external stress. Combined with reduced growth rates and metabolic specialization, these features favor the enrichment of tolerant and persister populations [9,21].

**Figure 3:**
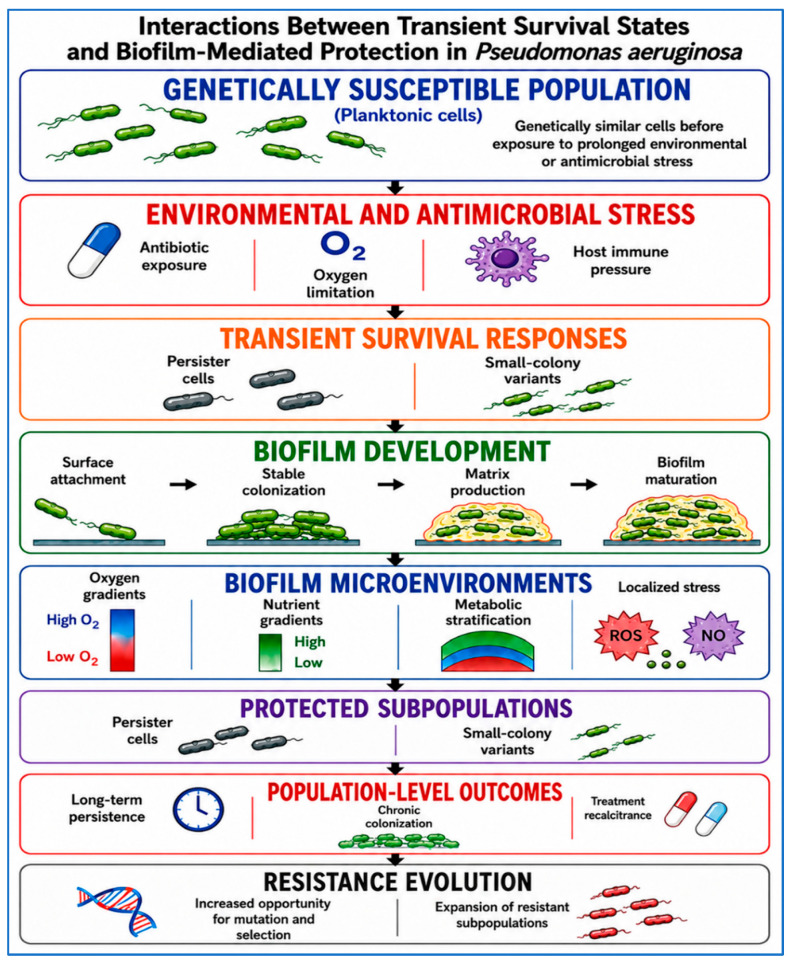
Interactions between transient survival programs and biofilm-mediated protection in *P. aeruginosa*. Environmental and antimicrobial stresses trigger reversible responses that enable bacterial populations to withstand unfavorable conditions. During biofilm growth, local variation in oxygen availability, nutrient access, and metabolic activity promotes the formation of protected subpopulations. These processes contribute to treatment recalcitrance, chronic colonization, and long-term persistence while increasing opportunities for resistance evolution.

Significantly, persistence and biofilm-mediated protection differ fundamentally from classical genetic resistance. Rather than supporting growth during antibiotic exposure, these mechanisms preserve viability during periods of stress. By maintaining bacterial populations throughout prolonged treatment and chronic infection, they increase the probability that resistant variants will eventually arise and become established. Persistence and biofilm formation therefore represent key intermediates linking short-term survival to the longer-term evolution of AMR [13,14]. The relationships among transient survival programs, biofilm-associated microenvironments, and population-level outcomes are summarized in Figure 3.

## 6. Collective Behavior and Ecological Success

The success of *P. aeruginosa* extends beyond its capacity to withstand stress and antimicrobial exposure. Equally important are population-level processes that support coordinated activity, efficient resource utilization, and occupation of diverse habitats. Through sophisticated communication networks and broad ecological versatility, *P. aeruginosa* inhabits a wide range of environmental and host-associated niches while retaining the ability to move between them. These characteristics contribute not only to environmental persistence but also to the dissemination of clinically important lineages [67,68,69].

### 6.1. Quorum Sensing and Collective Coordination

A defining characteristic of *P. aeruginosa* is its ability to coordinate population-wide activities through QS. Rather than functioning as isolated cells, bacterial populations communicate through diffusible signaling molecules that regulate gene expression according to population density and local ecological cues. This coordinated regulation promotes the synchronized production of extracellular enzymes, siderophores, biosurfactants, and other shared resources that benefit the community [36,70]. The role of QS extends beyond density-dependent communication. Environmental factors, including nutrient availability, spatial structure, host-derived signals, and light exposure, influence QS activity and shape community behavior [36,71]. Through this integration of multiple inputs, QS links communication networks with local ecological conditions and enables coordinated population responses.

The QS network of *P. aeruginosa* is organized into four interconnected systems: Las, Rhl, Pqs, and Iqs [19,37,38]. The Las system functions near the top of the regulatory hierarchy and controls the expression of many virulence-associated genes while influencing the activity of the Rhl and Pqs systems [37,38]. The Rhl system regulates the production of rhamnolipids, elastase, and other factors involved in biofilm development and host interaction [19,38]. The Pqs system contributes to biofilm maturation, stress adaptation, and intercellular communication through quinolone signaling molecules [38]. The Iqs system further integrates QS with environmental signals, allowing bacterial populations to adjust their behavior under changing conditions [36]. These interconnected systems coordinate collective behavior and enhance the adaptability of *P. aeruginosa* [19,36].

Recent studies have shown that signaling activity can vary among individual cells within the same population, producing temporary differences in communication states while preserving overall community organization [43]. This variability may enhance flexibility by allowing distinct cellular subgroups to contribute differently to collective functions. QS also plays a central role in cooperation. Many extracellular products synthesized by *P. aeruginosa* act as shared resources that improve nutrient acquisition, modify local habitats, or enhance competitive performance. However, cooperative systems create opportunities for exploitation by non-producing individuals, commonly referred to as social cheaters. Experimental studies have demonstrated that the balance between cooperation and cheating is strongly influenced by resource availability, spatial structure, and population composition [72,73,74]. Thus, QS serves not only as a communication network but also as a mechanism that regulates social behavior and maintains cooperative functions within bacterial communities.

### 6.2. Ecological Versatility and Environmental Connectivity

The widespread distribution of *P. aeruginosa* reflects an exceptional ability to colonize diverse environmental and host-associated habitats. The organism has been isolated from soil, freshwater systems, marine environments, plants, animals, wastewater systems, hospital infrastructures, and numerous human-associated niches [67,75]. This broad distribution is supported by extensive metabolic capabilities that allow utilization of diverse substrates and support survival under highly variable conditions.

Comparative genomic studies reveal that ecological success arises from the combination of a conserved core genome and a highly flexible accessory genome. Although many traits are shared among environmental and clinical populations, niche-specific genetic elements contribute to specialization within particular habitats. Environmental isolates often carry genes associated with chemotaxis, nutrient acquisition, environmental sensing, and alternative substrate utilization, whereas some clinical lineages show enrichment of determinants linked to host colonization and disease [68,76]. Phylogenetic analyses illustrate substantial overlap between environmental and clinical populations, suggesting continuous exchange across ecological boundaries rather than strict separation [67,76].

Environmental reservoirs play a key role in maintaining this connectivity. Rivers, sediments, wastewater systems, hydrocarbon-contaminated environments, agricultural ecosystems, and other aquatic habitats provide opportunities for long-term maintenance and dispersal [75,77,78]. Within healthcare settings, plumbing systems, sinks, faucets, and other water-associated infrastructures can function as persistent sources of survival and transmission [79,80,81]. These interconnected habitats facilitate movement of bacterial populations across environmental and clinical settings.

Connectivity also promotes genetic exchange. Plasmids, integrons, transposons, and integrative conjugative elements contribute to the dissemination of ecologically and clinically important traits among populations occupying shared habitats [68,76]. Consequently, environmental reservoirs serve not only as sites of maintenance but also as sources of genetic innovation that may influence the emergence of successful clinical lineages. The major habitats occupied by *P. aeruginosa*, together with their associated characteristics and potential contributions to resistance dynamics, are summarized in Table 2.

### 6.3. Ecological Consequences and One Health Implications

The broad ecological distribution of *P. aeruginosa* has important implications for environmental, veterinary, and human health. Continuous circulation among interconnected reservoirs creates opportunities for dissemination across geographic regions and habitat types. Long-term environmental maintenance increases the likelihood that biologically important traits will be retained within bacterial communities and transferred among populations through ecological interactions and genetic exchange [69].

The close relationship between environmental and clinical populations highlights the importance of a One Health perspective. Environmental habitats, wastewater systems, healthcare infrastructures, animals, and human hosts are best viewed as components of a connected ecological network rather than isolated compartments [68,69,82,83]. Changes occurring within one part of this network can influence bacterial populations elsewhere through transmission, selection, and evolutionary processes. This perspective has important implications for surveillance and control strategies. Interventions focused exclusively on clinical settings may overlook environmental reservoirs that contribute to long-term maintenance, dissemination, and genetic diversification [68,82]. Effective management therefore requires approaches that integrate environmental, veterinary, and clinical surveillance.

The ability of *P. aeruginosa* to coordinate collective behaviors, exploit diverse habitats, and circulate through interconnected reservoirs has contributed substantially to its global distribution and long-term success. These characteristics underscore the need for integrated monitoring and control strategies that account for both environmental and clinical dimensions of *P. aeruginosa* ecology. Long-term persistence within host-associated environments also creates opportunities for evolutionary change, a topic examined in the following section.

## 7. Evolutionary Change from Host Colonization to Population Dissemination

### 7.1. Evolution During Chronic Infection

The long-term survival of *P. aeruginosa* is closely linked to its capacity for rapid evolutionary change during infection. Following colonization, bacterial populations encounter host-specific pressures that promote continuous genetic diversification. Longitudinal studies have shown that this process often begins early and proceeds through the accumulation of mutations that improve performance within host-associated environments [84,85,86]. Rather than remaining genetically uniform, chronic populations frequently diversify into multiple coexisting sublineages. Whole-genome sequencing studies have revealed extensive within-host diversity, with independent lineages acquiring distinct mutations while occupying the same infection site [54,87,88]. This diversification broadens the range of phenotypes within the population and increases the likelihood that some variants remain successful under changing host conditions.

A notable feature of chronic infection is the repeated appearance of similar mutations in unrelated lineages. Genes involved in global regulation, including *mucA*, *lasR*, and *rpoN*, are among the most frequent evolutionary targets, providing strong evidence of convergent evolution across different hosts [25,54,84,86]. Because these regulators influence multiple cellular functions, mutations in these loci can simultaneously alter metabolism, stress responses, QS, and virulence-associated activities.

Recent population genomic studies demonstrate that chronic infections often contain polymorphic populations composed of multiple coexisting variants rather than a single dominant genotype [89]. The presence of genetically distinct subpopulations permits parallel evolutionary pathways within the same host. Large-scale comparative genomic analyses have also identified hundreds of recurrent pathoadaptive genes associated with host-associated evolution, indicating that genetic change frequently follows predictable patterns rather than occurring at random [49]. These findings exhibit that within-host evolution is characterized by diversification, convergence, and the progressive accumulation of traits that improve performance within host environments.

### 7.2. Host-Driven Selection and the Emergence of Successful Variants

Although genetic variation arises continuously during infection, only a fraction of variants becomes established within bacterial populations. Their success is determined by selective pressures imposed by the host, including immune activity, oxidative stress, nutrient limitation, oxygen restriction, and antimicrobial exposure [85,90,91]. Among the most common outcomes is a shift from phenotypes associated with acute infection toward traits that favor long-term colonization. Mucoid variants generated through mutations in *mucA* provide increased protection against host defenses, whereas mutations affecting *lasR* are associated with metabolic remodeling and improved performance in chronic infection environments [85,90,92]. The repeated selection of these mutations across independent infections indicates that they confer substantial advantages under prolonged host-associated selection.

Selection may also favor hypermutable lineages. Mutations affecting DNA repair pathways, particularly *mutS* and *mutL*, increase mutation rates and accelerate the generation of genetic diversity [85,90]. Although hypermutability can impose biological costs, it also increases the probability of acquiring beneficial mutations, including those associated with AMR. Antibiotic treatment represents one of the strongest selective forces encountered during infection. Longitudinal genomic studies have shown that resistant variants can emerge rapidly during therapy and expand when resistance-associated benefits outweigh fitness costs [93]. However, these trajectories are rarely linear and often reflect ongoing competition among coexisting lineages together with changing host conditions.

The specific host niche also influences evolutionary outcomes. Comparative genomic analyses have identified distinct genetic signatures in isolates recovered from chronic respiratory infections and bloodstream infections, indicating that different host environments favor different combinations of metabolic, stress-response, and virulence-associated characteristics [49,94]. These findings illustrate how host-associated selection shapes populations that become increasingly specialized for particular infection settings.

### 7.3. Clonal Expansion, Host Specialization, and Population Dissemination

Evolution within individual hosts provides the foundation for broader population success. In some cases, traits that arise during infection become established within lineages that subsequently spread beyond their original host and contribute to the emergence of epidemic clones. Population genomic studies indicate that *P. aeruginosa* exhibits a predominantly nonclonal epidemic population structure characterized by extensive diversity and frequent recombination [91,95]. Despite this diversity, a limited number of highly successful lineages contribute disproportionately to the global burden of multidrug-resistant infection.

Among the most important are the internationally distributed high-risk clones ST235, ST111, and ST175 [96,97]. Their success reflects the combined effects of chromosomal mutations, horizontally acquired resistance determinants, and accessory genomic elements that improve survival under antimicrobial pressure. Different lineages have followed distinct evolutionary routes, including the acquisition of mobile genetic elements and chromosomal modifications affecting porin expression, efflux systems, and β-lactamase regulation [96,98].

Large-scale genomic studies indicate that repeated selection within particular host environments can generate specialized lineages with distinct ecological characteristics, transmission potential, and clinical behavior [49,94]. Such specialization contributes to the emergence of successful epidemic clones. The spread of high-risk lineages is further facilitated by the broad ecological distribution of *P. aeruginosa*. These clones have been identified in healthcare settings, wastewater systems, urban environments, animals, and other environmental reservoirs, underscoring the interconnected nature of environmental and clinical populations [97,99].

**Figure 4:**
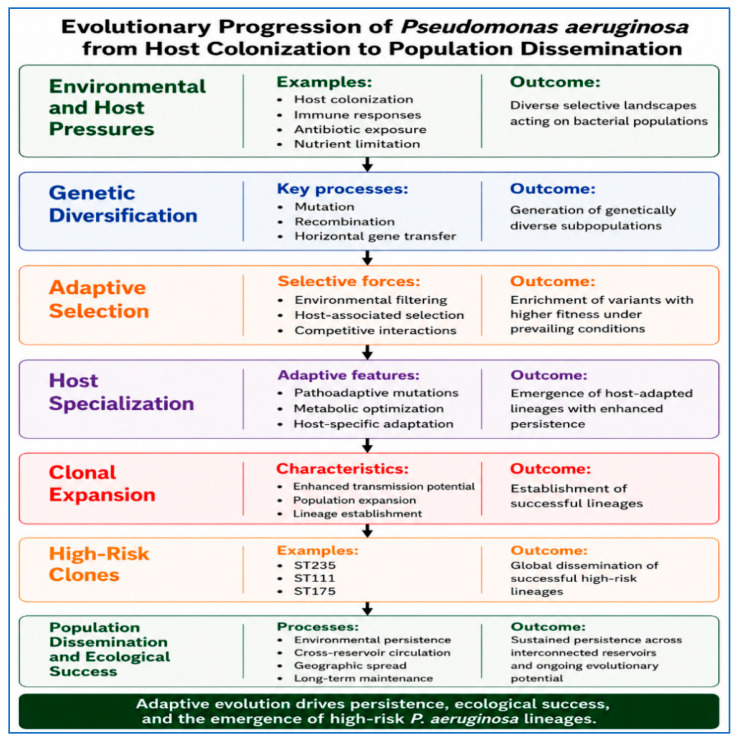
Evolutionary progression of *P. aeruginosa* from host colonization to population dissemination. Host-associated and environmental pressures promote genetic diversification, selection, and specialization, ultimately leading to clonal expansion and the emergence of high-risk lineages. The persistence and spread of these lineages across interconnected reservoirs contribute to the long-term ecological success of *P. aeruginosa*.

These findings reveal that evolution in *P. aeruginosa* is a continuous process linking within-host diversification, lineage specialization, and population dissemination. Through the combined effects of mutation, selection, diversification, and transmission, bacterial populations continually generate and refine traits that contribute to long-term survival, ecological success, and the emergence of globally distributed high-risk clones. These outcomes illustrate how evolutionary processes shape the resistance phenotypes, persistence mechanisms, and ecological patterns discussed throughout this review. The progression from within-host diversification to population dissemination is summarized in Figure 4.

## 8. Toward a Systems-Level Understanding of Resistance in *P. aeruginosa*

### 8.1. Why Resistance Cannot Be Explained by Resistance Genes Alone

AMR in *P. aeruginosa* has traditionally been interpreted through the acquisition and expression of resistance determinants, including efflux systems, porin modifications, target-site alterations, and horizontally acquired resistance genes [7,96]. Although these mechanisms remain central to AMR, they do not fully explain the ability of this pathogen to survive antimicrobial treatment, persist within hosts, and establish chronic infections [8,16]. Evidence reviewed throughout this article indicates that bacterial survival is also shaped by processes that extend beyond stable genetic resistance. Phenotypic plasticity, population heterogeneity, persistence, tolerance, and biofilm-mediated protection enable bacterial populations to withstand antimicrobial exposure under circumstances where resistance determinants alone cannot account for treatment outcomes [9,13,14].

Resistance should therefore not be viewed solely as a genetic characteristic. Instead, it represents one component of a broader biological response shaped by interactions among cellular physiology, population structure, ecological context, and evolutionary change [8,16]. This distinction has important clinical implications. Antimicrobial susceptibility testing primarily evaluates genetically encoded resistance under standardized laboratory conditions, whereas bacterial populations during infection experience fluctuating host environments characterized by immune pressure, nutrient limitation, oxygen gradients, microbial interactions, and repeated antimicrobial exposure [8,16]. These factors can substantially influence survival without altering resistance genotypes. Subsequently, understanding treatment failure requires consideration of processes operating across multiple levels of biological organization rather than exclusive focus on resistance genes.

### 8.2. Resistance as an Emergent System Property

Viewing *P. aeruginosa* as a complex adaptive system provides a broader framework for knowing the origin and persistence of resistance. Within this framework, resistance is not a single phenotype generated by an isolated mechanism but a system-level property arising from interactions among regulatory networks, cellular physiology, microbial communities, ecological pressures, and evolutionary processes [8,30]. Environmental and host-associated challenges continuously influence bacterial behavior through interconnected regulatory pathways that coordinate cellular responses. These processes can promote phenotypic diversification, alter metabolic activity, stimulate biofilm development, and support the formation of persistent subpopulations. As a result, bacterial populations become composed of functionally distinct cells that differ in growth, stress tolerance, and survival potential [23,25].

Survival itself can influence future evolutionary outcomes. Persistent and tolerant subpopulations maintain viable reservoirs during antimicrobial treatment, increasing opportunities for mutation, selection, and lineage diversification. Repeated cycles of stress, survival, and selection may subsequently promote the emergence of highly successful lineages capable of long-term persistence and dissemination [14,49,54]. These relationships are reinforced by feedback loops operating across multiple organizational levels. Antimicrobial exposure can enrich persistent phenotypes, persistence can increase opportunities for evolutionary change, and evolutionary change can generate populations with improved survival under future selective pressures. Resistance therefore arises from the cumulative effects of interacting physiological, ecological, and evolutionary processes rather than from individual resistance determinants alone [8,21,49,100].

**Figure 5:**
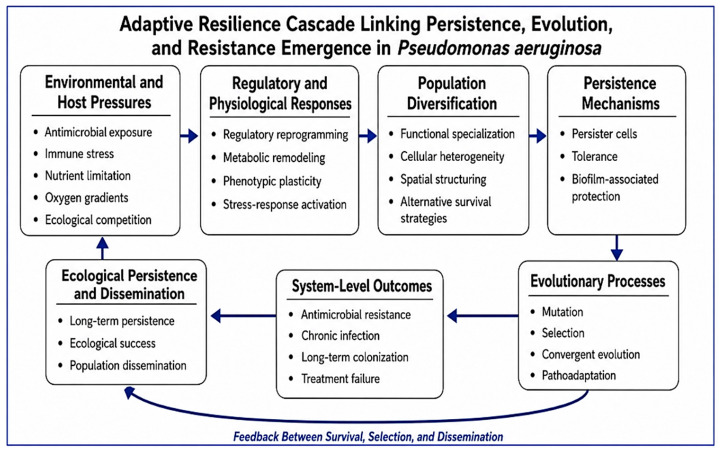
Adaptive resilience cascade illustrating how environmental sensing, physiological diversification, persistence, evolutionary change, and ecological dissemination interact through interconnected feedback loops to generate resistance and long-term survival in *P. aeruginosa*. The framework proposes that processes operating across cellular, population, ecological, and evolutionary levels collectively shape persistence, dissemination, and resistance emergence.

The evidence reviewed here supports a shift from a resistance-centered model toward a systems-oriented model of AMR. In this view, resistance genes are important but represent only one outcome of broader processes that include environmental sensing, phenotypic plasticity, population diversification, persistence, and ecological selection. Understanding how these processes interact may be as important as identifying resistance determinants themselves. Figure 5 summarizes these relationships through the proposed adaptive resilience cascade, which links environmental sensing, physiological diversification, persistence, evolutionary change, and dissemination within a unified explanatory framework.

### 8.3. Implications for Surveillance, Therapeutics, and Stewardship

Recognizing *P. aeruginosa* as a dynamic biological system has important implications for AMR surveillance. Conventional surveillance programs focus primarily on resistance phenotypes and resistance genes. Although these approaches remain essential, they may not fully capture traits that contribute to persistence, transmission, and treatment failure [16,68]. Integrating genomic, ecological, and longitudinal surveillance strategies can provide a more complete understanding of how resistance emerges and spreads within interconnected populations [49,69].

Current AMR surveillance is highly effective at identifying resistance genes and resistance phenotypes but is less able to detect adaptive processes that do not involve stable genetic changes. As a result, important mechanisms such as persistence, phenotypic heterogeneity, and biofilm-associated survival may remain undetected despite their major contribution to treatment failure. Integrating genomic, transcriptomic, phenotypic, and ecological data can provide a more complete understanding of bacterial behavior, improve surveillance, and support earlier identification of infections with a high risk of persistence or poor clinical outcomes [16,30,44,69].

From a clinical perspective, *P. aeruginosa* remains one of the most challenging bacterial pathogens because its ability to cause persistent and recurrent infections extends beyond AMR alone. Biofilm formation, phenotypic heterogeneity, persistence, and adaptive responses work together to reduce treatment effectiveness and contribute to chronic infections, prolonged hospitalization, and poor clinical outcomes [9,13,14,16]. Understanding these interconnected processes may support earlier intervention, more effective treatment strategies, and improved antimicrobial stewardship.

Routine diagnostic tests can reliably identify genetically encoded AMR but are much less effective at detecting transient phenotypic states such as tolerance and persistence. As a result, bacterial populations that appear susceptible in laboratory testing may still survive treatment and contribute to recurrent infections. Combining conventional susceptibility testing with molecular methods, single-cell technologies, and other advanced phenotypic approaches may improve the detection of these adaptive states and support more informed treatment decisions in the future [16,44].

This perspective also has implications for therapeutic development. Interventions directed exclusively toward resistance determinants may leave other survival mechanisms unaffected. Increasing evidence indicates that persistence, biofilm-mediated protection, stress-response pathways, and population heterogeneity contribute substantially to chronic infection and treatment failure, suggesting that these processes represent important complementary therapeutic targets [9,13,14].

Antimicrobial stewardship may likewise benefit from a systems-oriented perspective. Beyond minimizing the selection of resistant mutants, stewardship programs should consider how treatment strategies influence population structure, ecological relationships, and evolutionary trajectories [8,16]. Approaches that reduce prolonged selective pressure, limit opportunities for persistence, and constrain lineage diversification may contribute to more sustainable resistance management.

These observations support a transition from a predominantly gene-centered view of AMR toward an integrative framework that incorporates physiology, population biology, ecological interactions, and evolutionary change. Such a perspective provides a more realistic foundation for understanding, monitoring, and ultimately controlling AMR in *P. aeruginosa*.

## 9. Future Perspectives: Disrupting System-Level Survival Processes Rather than Resistance Genes

### 9.1. Targeting Population-Level Survival Mechanisms

Future strategies against *P. aeruginosa* are likely to extend beyond the traditional focus on resistance genes and increasingly target the population-level processes that support persistence, specialization, and long-term survival. Recent single-cell studies have revealed extensive heterogeneity within genetically identical populations, challenging the longstanding view of *P. aeruginosa* as a functionally uniform pathogen [16,101]. Even genetically identical cells can adopt distinct functional programs, generating specialized subpopulations that contribute differently to virulence, communication, and survival [101].

Single-cell imaging-transcriptomic analyses have identified substantial variation in QS activity among individual cells, including signaling-primed and hypersignaling subpopulations [101]. These observations suggest that a relatively small fraction of cells may exert disproportionate influence over population-wide behavior. Future interventions may therefore focus on disrupting key cellular programs before they coordinate community-level responses.

Virulence expression is also unevenly distributed across bacterial populations. Using single-cell approaches, Chen et al. [102] exhibited that cyclic-di-GMP promotes the formation of distinct subpopulations characterized by different lifestyles and virulence programs. This division of labor appears to enhance population performance under changing conditions. Therefore, future therapies may target the regulatory circuits that generate these specialized subgroups, reducing the ability of bacterial populations to withstand host defenses and antimicrobial treatment.

Quantitative systems biology and mathematical modeling will also be important for advancing this field. By integrating experimental data with computational models, future studies may estimate the relative contributions of environmental sensing, phenotypic plasticity, persistence, and evolutionary adaptation to bacterial survival and AMR [30,44]. Such approaches could strengthen the predictive value of the proposed adaptive resilience cascade and support the development of more targeted therapeutic strategies [102].

### 9.2. Ecological and Evolutionary Interventions

The success of *P. aeruginosa* is closely linked to its ability to occupy diverse habitats and respond rapidly to selective pressures [16]. Future control strategies may therefore focus not only on bacterial eradication but also on influencing the evolutionary pathways available to bacterial populations [103].

Several emerging therapeutic strategies are being explored to improve the treatment of multidrug-resistant *P. aeruginosa*. These include bacteriophage therapy, engineered phages, pyocins, and combination therapies designed to enhance antibiotic activity while limiting resistance development [104,105,106,107,108,109,110,111]. Although many of these approaches remain under investigation, they represent promising alternatives to conventional antibiotics for managing persistent and drug-resistant infections.

Bacteriophage-based therapies represent one of the most promising examples of this approach. Unlike conventional antibiotics, bacteriophages can evolve alongside bacterial populations. Recent advances in phage engineering have enabled the development of customized lytic phages with improved host specificity and therapeutic potential [104]. These technologies offer opportunities to target bacterial populations while maintaining activity against newly emerging variants.

Phage resistance nevertheless remains a significant challenge. Studies have shown that *P. aeruginosa* can rapidly develop mechanisms that reduce susceptibility to phage infection [105]. To address this limitation, current approaches increasingly employ phage cocktails, engineered phages, and phage-antibiotic combinations designed to reduce opportunities for evolutionary escape [106,107].

Another promising direction involves exploiting evolutionary trade-offs. Adaptation to one selective pressure may reduce performance under another, creating opportunities to direct bacterial evolution toward more treatable phenotypes. Experimental studies have shown that resistance to bacteriophages or specific antibiotics may be accompanied by reduced virulence, impaired fitness, or renewed susceptibility to antimicrobial agent [108,109,110]. A deeper understanding of these trade-offs could allow clinicians to steer bacterial populations toward states that are less virulent, less persistent, or more responsive to treatment. Although these approaches remain at an early stage of development, they provide a framework for influencing evolutionary trajectories rather than simply opposing them [103].

Pyocins offer a complementary ecological strategy. These narrow-spectrum bacteriocins can selectively eliminate susceptible *P. aeruginosa* strains while minimizing disruption of surrounding microbial communities. Recent studies demonstrating effective multi-pyocin formulations highlight their potential as precision antimicrobials for difficult-to-treat infections [111]. Instead of focusing solely on eradication, future ecological interventions may seek to reshape population structure, restrict evolutionary opportunities, and reduce the long-term spread of highly successful lineages.

Current evidence suggests that no single strategy is likely to provide durable control of *P. aeruginosa*. Instead, combination approaches that target complementary adaptive processes may offer the greatest clinical potential. Among the available strategies, phage-antibiotic combinations have shown particular promise because they may reduce the emergence of resistant mutants while preserving treatment effectiveness [106,107]. Approaches that interfere with quorum sensing or cyclic-di-GMP signaling may enhance these therapies by reducing virulence and biofilm formation rather than directly selecting for resistance. From a stewardship perspective, treatment strategies should consider persistence and treatment duration in addition to AMR, with the goal of limiting the survival of persistent bacterial subpopulations while maintaining effective infection control [8,16,103].

### 9.3. Systems-Based Approaches for Future Amr Control

The complexity of *P. aeruginosa* requires approaches capable of capturing processes that operate from individual cells to host-associated ecosystems. Recent advances in spatial transcriptomics are beginning to provide this level of resolution. By simultaneously mapping bacterial and host gene expression within infected tissues, [112] identified localized host-pathogen interactions and uncovered previously unrecognized virulence-associated genes. These findings demonstrate how spatially resolved analyses can reveal biological vulnerabilities that remain hidden in conventional laboratory models and may support the development of more precise intervention strategies.

Rapid advances in single-cell multi-omics, spatial multi-omics, long-read sequencing, and real-time analysis of host-pathogen interactions are expected to provide a more detailed understanding of bacterial evolution during infection. As these technologies mature and larger longitudinal datasets become available, they are likely to reveal adaptive processes that cannot be detected using conventional population-based approaches and may improve prediction of within-host evolutionary trajectories [101,112,113,114].

Artificial intelligence is also becoming an important tool for understanding *P. aeruginosa*. Recent computational frameworks have demonstrated the ability to predict strain-specific metabolic capabilities and uncover previously unrecognized physiological traits across diverse populations [113]. In addition, explainable machine-learning models have been developed to predict AMR directly from genomic sequences, providing biologically interpretable insights into resistance-associated signatures and improving phenotype prediction [114]. As genomic, transcriptomic, proteomic, and metabolic datasets continue to expand, machine-learning approaches may improve prediction of bacterial behavior and support more individualized treatment strategies.

Advances in biological sensing technologies are expected to strengthen future surveillance systems. Engineered reporter bacteriophages capable of rapidly detecting viable *P. aeruginosa* cells illustrate how next-generation diagnostics may move beyond simple resistance profiling toward real-time monitoring of bacterial activity and infection dynamics [115]. Despite these advances, important challenges remain. Processes that contribute to persistence and treatment failure vary considerably among infection types, host environments, and ecological settings. No single framework can fully capture all dimensions of *P. aeruginosa* biology, and the relative contribution of individual mechanisms is likely to differ among strains, infection sites, and stages of disease progression.

Although regulatory genes such as *mucA* and *lasR* are well recognized for their role in adaptation during chronic lung infection, it remains unclear whether similar regulatory nodes consistently shape evolution across other infection sites. Identifying conserved adaptive pathways across diverse clinical and environmental settings will require larger comparative studies. Another important challenge is integrating ecological and clinical datasets, which often differ in sampling methods, study design, and data collection. Improved integration of these complementary data sources will strengthen our understanding of bacterial adaptation across interconnected reservoirs and support a broader One Health perspective [16,49,69].

Ultimately, effective management of *P. aeruginosa* may depend on recognizing resistance as one component of a broader biological system [16]. Integrating single-cell biology, evolutionary principles, ecological engineering, synthetic biology, and computational modeling offers opportunities to identify vulnerabilities that are not apparent when resistance genes are examined in isolation [16,103]. The next generation of antimicrobial strategies may therefore focus on disrupting the organizational processes that sustain survival across multiple levels of biological organization. Nevertheless, many of these approaches remain experimental, and their clinical effectiveness, scalability, and long-term evolutionary consequences require further investigation.

## 10. Limitations of Current Evidence

Despite considerable advances in understanding the adaptation and AMR of *P. aeruginosa*, several important limitations remain in the available literature. Much of the current evidence is derived from laboratory-based or in vitro studies that cannot fully reproduce the complexity of human infections. In addition, many studies investigate individual mechanisms, such as biofilm formation, quorum sensing, persistence, or resistance, independently rather than examining how these processes interact across multiple biological scales.

Another important limitation is that a large proportion of the available evidence originates from chronic respiratory infection models, particularly cystic fibrosis, which may limit the generalizability of these findings to other infection sites and clinical settings. Although recent advances in single-cell analysis, spatial omics, and multi-omics technologies are providing new insights into bacterial adaptation, these approaches remain relatively underused and have not yet been widely integrated into microbiological or clinical studies.

Finally, the adaptive resilience cascade proposed in this review should be regarded as a conceptual framework that integrates current evidence rather than as a validated predictive model. Future experimental, clinical, and longitudinal studies will be necessary to evaluate this framework, clarify the interactions among adaptive processes, and determine its value for improving antimicrobial surveillance and therapeutic development.

## 11. Conclusions

*P. aeruginosa* remains a major clinical challenge because its success is driven by a highly integrated biological system that extends far beyond the acquisition of resistance genes. Environmental sensing, phenotypic plasticity, population diversification, persistence, biofilm-associated protection, and evolutionary change collectively enable this pathogen to survive antimicrobial exposure, establish chronic infections, and occupy a wide range of environmental and host-associated niches. The evidence reviewed here indicates that resistance is only one component of a broader network of interconnected processes that shape bacterial survival and long-term success. Viewing *P. aeruginosa* through a complex adaptive systems framework provides a more comprehensive explanation for treatment failure, chronic colonization, and resistance emergence than gene-centered models alone. Rather than arising from isolated genetic determinants, these outcomes reflect interactions among regulatory networks, physiological responses, population structure, ecological pressures, and evolutionary processes operating across multiple levels of biological organization. A central contribution of this review is the proposed adaptive resilience cascade, which integrates environmental sensing, physiological diversification, persistence mechanisms, evolutionary change, and dissemination within a unified conceptual framework. This perspective highlights how processes occurring at cellular, population, ecological, and evolutionary levels collectively shape resistance emergence and long-term survival. Future efforts to control *P. aeruginosa* will benefit from strategies that extend beyond resistance determinants and address the broader processes that sustain persistence, diversification, and dissemination. Integrating ecological, evolutionary, physiological, and systems-level perspectives may provide a stronger foundation for surveillance, therapeutic development, and antimicrobial stewardship. Ultimately, understanding resistance as a system-level outcome rather than an isolated genetic trait may be essential for developing more effective and sustainable approaches to combating AMR in *P. aeruginosa*.

## Figures and Tables

**Table 1:** Environmental signals, cellular responses, and ecological consequences in *P. aeruginosa*.

Environmental Signal	Primary Cellular Response	Ecological Consequence	Refs.
Oxygen limitation	Alternative respiratory metabolism and denitrification	Persistence under hypoxic conditions	[60]
Iron restriction	Enhanced siderophore-mediated iron acquisition	Maintenance of growth and virulence under iron scarcity	[61]
Oxidative stress	Activation of antioxidant defense systems	Protection against reactive oxygen species	[62]
Surface association	Biofilm-associated physiological transition	Enhanced biofilm establishment and persistence	[63]
Biofilm microenvironmental gradients	Formation of spatially differentiated cellular subpopulations	Functional heterogeneity within microbial communities	[52]
Localized physicochemical variation	Metabolic specialization of subpopulations	Spatial metabolic specialization within biofilms	[51]
Antimicrobial exposure	Transient tolerance-associated phenotypes	Short-term survival during treatment	[48]

**Table 2:** Major Ecological Niches of *P. aeruginosa* and Their Potential Contributions to Resistance Dynamics.

Ecological Niche	Key Ecological Characteristic	Ecological Significance	Contribution to Resistance Dynamics	Refs.
Freshwater systems and rivers	Broad metabolic capacity	Long-term environmental persistence and dispersal	Maintenance and dissemination of adaptive populations	[67,75]
River sediments and aquatic interfaces	Surface-associated growth	Stable environmental reservoir	Persistence of diverse bacterial populations	[75,77]
Wastewater systems	High microbial diversity and genetic exchange	Environmental mixing hub	Facilitates horizontal transfer of ecologically relevant traits	[69]
Agricultural environments	Survival across variable habitats	Colonization of plant-associated habitats	Exposure to environmental selective pressures	[75,78]
Hydrocarbon-contaminated habitats	Utilization of diverse substrates	Survival in polluted ecosystems	Selection of environmentally successful lineages	[75]
Hospital water systems	Long-term colonization of water-associated infrastructure	Environmental-clinical connectivity	Source of transmission and dissemination of clinically relevant strains	[79,80,81]
Environmental and clinical niches	Accessory genome variability	Niche adaptation and ecological expansion	Emergence of successful high-risk lineages	[68,76]
Interconnected environmental reservoirs	Mobile genetic elements	Evolutionary diversification	Dissemination of resistance determinants	[68,69,76]

## Data Availability

No new data were created or analyzed in this study.
